# On the reliability of seasonal climate forecasts

**DOI:** 10.1098/rsif.2013.1162

**Published:** 2014-07-06

**Authors:** A. Weisheimer, T. N. Palmer

**Affiliations:** 1 Department of Physics, National Centre for Atmospheric Science (NCAS), University of Oxford, Oxford OX1 3PU, UK; 2 European Centre for Medium-Range Weather Forecasts (ECMWF), Reading RG2 9AX, UK

**Keywords:** climate, forecasts, reliability, seasonal

## Abstract

Seasonal climate forecasts are being used increasingly across a range of application sectors. A recent UK governmental report asked: how good are seasonal forecasts on a scale of 1–5 (where 5 is very good), and how good can we expect them to be in 30 years time? Seasonal forecasts are made from ensembles of integrations of numerical models of climate. We argue that ‘goodness’ should be assessed first and foremost in terms of the probabilistic reliability of these ensemble-based forecasts; reliable inputs are essential for any forecast-based decision-making. We propose that a ‘5’ should be reserved for systems that are not only reliable overall, but where, in particular, small ensemble spread is a reliable indicator of low ensemble forecast error. We study the reliability of regional temperature and precipitation forecasts of the current operational seasonal forecast system of the European Centre for Medium-Range Weather Forecasts, universally regarded as one of the world-leading operational institutes producing seasonal climate forecasts. A wide range of ‘goodness’ rankings, depending on region and variable (with summer forecasts of rainfall over Northern Europe performing exceptionally poorly) is found. Finally, we discuss the prospects of reaching ‘5’ across all regions and variables in 30 years time.

## 1. Introduction

Over the past 30 years, the science of predicting seasonal-timescale fluctuations in weather has grown from a research activity undertaken in a few academic and research institutes [1], to a routine operational activity in a number of meteorological forecast services [2–4]. Unlike conventional weather forecasts, seasonal predictions do not attempt to forecast the detailed day-to-day evolution of weather. Such detailed prediction is ruled out by the chaotic nature of the climate system, otherwise known as the ‘butterfly effect’ [5]. Rather, seasonal predictions provide estimates of seasonal-mean statistics of weather, typically up to three months ahead of the season in question. Hence, for example, a seasonal forecast can provide information on how likely it is that the coming season will be wetter, drier, warmer or colder than normal. The physical basis for such estimates arises from the effect of predictable seasonal-timescale signals arising from the ocean, and to a lesser extent the land surface, on the atmosphere [6]. The key paradigm for seasonal forecasting is El Niño, a coupled ocean–atmosphere phenomenon occurring primarily in the tropical Pacific and predictable six months and more ahead [7,8].

Such information is relevant to a variety of users in weather-sensitive sectors, and therefore can influence decisions made in these sectors. As a result, seasonal climate forecasts are increasingly being used across a range of application areas [9]. For example, information about seasonal average rainfall and temperature for the growing season can potentially influence a farmer's decision about which crops to plant ahead of time, or a humanitarian organization's strategy for anticipating food shortages in drought-prone regions of the developing world. However, this information is only useful if it is skilful.

In the literature, there exists a plethora of methods to estimate the skill of forecasts [10]. In general, each of these methods quantifies a different detailed aspect of the forecast quality. In this paper, however, we try to simplify the question. Rather than coming up with complex estimates of different characteristics of forecast skill whose relevance strongly depends on the specific application, we simply ask: on a scale of 1–5, where 5 is very good, how skilful are seasonal forecasts today? On a similar scale, how skilful can we expect seasonal forecasts to be 30 years from now? These types of question are sufficiently open-ended that they may appear impossibly difficult to answer in any such succinct way. And yet precisely, these types of question are being asked by policymakers, e.g. by the UK Government as it considers options for future investment in science [11].

What level of skill should a seasonal forecast system reach, to merit being rated a ‘5’? Would every forecast of seasonal-mean temperature and rainfall for a particular region have to be both precise and accurate? This is likely to be an impossible goal. While the impact of the butterfly effect is mitigated substantially by focusing on prediction of seasonal-mean rather than instantaneous variables, it is not eliminated entirely. In addition, the coupled ocean–atmosphere models used to make seasonal forecasts are finite truncations of the underlying (partial differential) equations of the climate system, and hence are only approximate representations of reality. These two facts of the matter imply that seasonal predictions must necessarily be considered probabilistic in character—forecasts from any deterministic seasonal prediction will necessarily be unreliable and therefore untrustworthy.

However, this does not imply that any probabilistic forecast system is necessarily reliable. In this paper, we define a forecast as being reliable if it demonstrates statistical reliability in the following sense. Consider a set of predictions derived from ensemble forecasts. For some of these cases, it is predicted that the chance of above-average seasonal-mean rainfall for the coming growing season will be 80%. If the probabilistic forecast system is reliable, then one can expect that in 80% of these predictions the actual seasonal-mean rainfall will be above average. In this way, the concept of ‘reliability’ can be extended to probabilistic forecast systems [12]. If a forecast system is unreliable in this sense, then poor decisions can be made. As discussed in §2, a farmer might decide that it makes economic sense to grow a particular type of crop when the forecast for above-average rain exceeds 80%. However, if, in reality, above-average rain only occurs 50% of the time when the forecast probability exceeds 80%, then the potential economic benefit of planting the particular crop may be completely lost by the unreliable probabilistic forecast.

This raises an important conceptual point. Consider a hypothetical forecast system which most of the time forecasts climatological probabilities, but occasionally forecasts probabilities that are substantially different from climatology. If the probability forecasts have reliability and if the system can successfully discriminate between predictable and unpredictable situations, then we would rate such a system as ‘5’ even though the formal skill scores such as the Brier skill score (see below) for such a system may be small. We can compare this with an ensemble forecast system where the forecast probabilities are comparable with climatology for all initial conditions. Here, we would rate such a system as ‘2’—such a forecast system would never lead decision-makers to make poor decisions, though it might not be particularly useful.

In this paper, we develop objective criteria for classifying forecast skill into five categories, discuss how close we are to achieving a ‘5’ today, and consider what is needed to achieve a ‘5’ in 30 years time. All results presented here are based on the (state-of-the-art) operational seasonal forecast System 4 from the European Centre for Medium-Range Weather Forecasts (ECMWF). Depending on the region and variable being studied, we find examples of all five of our categories.

## 2. Probabilistic skill and decision-making

Forecasts are used to make decisions. For example, a farmer wants to decide what type of crop to plant in the coming season. Suppose there is a choice between two types of crop: *A* and *B*. The crop yield (tonnes per hectare) *C_A_* and *C_B_* of *A* and *B* depends on a number of meteorological variables such as temperature and precipitation, collectively labelled by *X.* Hence, *C_A_* = *C_A_*(*X*) and *C_B_* = *C_B_*(*X*). Suppose we have a forecast system that predicts over a given season a probability distribution *ρ*(*X*) for *X.* Then, the expected crop yield for *A* and *B* is
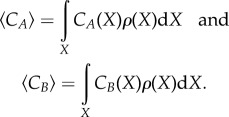
If 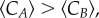 then the farmer might choose *A* over *B,* and vice versa. In practice of course, there will be many factors other than climate that determine the farmer's decision, e.g. details of the distributions *C_A_* and *C_B_*, but let us suppose here that climate is the only relevant one.

In general, one can expect *C_A_* to be a nonlinear function of *X*. Hence, 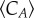 depends on more than just the mode of the distribution *ρ*. The uncertainty, given by the spread of the forecast distribution, might have just as large an impact on the estimate 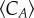 as does the mode of the forecast distribution.

Let us assume that
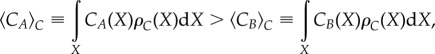
where *ρ_C_*(*X*) is the climatological distribution of *X*. Let us also suppose that in the majority of forecast occasions, the forecast distribution *ρ*(*X*) is not significantly different from the climatological distribution *ρ_C_*(*X*). Then, on these occasions, while the farmer is not going to gain any specially useful information from the forecast system, (s)he is not going to be misled by unreliable information. Conversely, consider the relatively infrequent occasions where 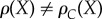 such that 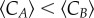. If as a result the farmer decides to grow *B* over *A*, then it is essential that the forecast probability function *ρ*(*X*) must be reliable.

One way to assess whether such forecast distributions *ρ* are reliable when 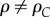 is to study so-called attributes (or ‘reliability’) diagrams. Reliability diagrams are discussed and shown in §§4 and 5. The focus if this paper is the reliability of user-relevant forecast variables in ECMWF's System 4 seasonal forecasts in the situations where 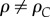.

## 3. The European Centre for Medium-Range Weather Forecasts seasonal forecast System 4

The ECMWF has been at the forefront of seasonal predictions for many years. Research on predictability on seasonal timescale in the early 1990s [6] led to the implementation of the first ECMWF seasonal forecast system based on a global ocean–atmosphere coupled model in 1997, and a successful forecast of the major 1997–1998 El Niño [13]. This first coupled System 1 was replaced by System 2 in 2001 and System 3 in March 2007. In November 2011, the latest seasonal forecasting System 4 started producing operational forecasts. The results presented in this paper are based on System 4's retrospective seasonal forecasts of 2 m temperature and precipitation over land.

The forecasting model of System 4 [14] consists of an atmospheric and an oceanic component to simulate the evolution of the global circulation in the atmosphere and in the oceans, based on the physical laws of fluid dynamics. The equations of motions and the thermodynamic laws are solved numerically by discretizing the atmosphere and the oceans into several vertical layers and horizontal grid boxes. The atmospheric model component of System 4 is version CY36R4 of ECMWF's weather forecasting model IFS (Integrated Forecasting System). Although the model for ensemble weather forecasting is run with a horizontal resolution of approximately 30 km-sized grid boxes, the resolution used in seasonal forecasting of approximately 80 km (spectral resolution of T255) is somewhat coarser. The atmospheric model has 91 vertical levels reaching up to 0.01 hPa. The ocean model used in System 4 is NEMO (Nucleus for European Modelling of the Ocean) version 3.0, a state-of-the-art modelling framework for oceanic research. The ocean model has 42 levels in the vertical, and the grid boxes have an approximately length of 110 km (1°).

As discussed in the Introduction, seasonal forecasts must be probabilistic by nature. In practice, probabilistic forecasts are derived by running an ensemble of integrations of the forecast model. Each member of the ensemble uses slightly different initial conditions and different realizations of stochastic representations of subgrid physical processes in the atmosphere. Operational global forecasts with System 4 are produced at the beginning of each month for forecast lead times of seven months into the future using 51 ensemble members.

How can we estimate the reliability of these seasonal forecasts? A single probabilistic forecast cannot, in general, be verified or falsified. But, for a set of probabilistic forecasts, we can evaluate the performance of the forecasting system by systematically comparing the forecasts with observations and by deriving statistical skill measures. These skill estimates, based on the performance of the system in the past, may guide users about the expected performance of the forecasts of the future. With this paper, however, we do not aim to analyse the probabilistic forecast skill of System 4 as this has been done elsewhere [14–16]. Rather, we focus on the reliability of the forecasts, as discussed in the Introduction and §4. A caveat with estimating skill from the past is the non-stationary quality of the observations available owing to changes in the observational system.

In order to achieve a robust estimate of the System 4 model performance, an extensive set of retrospective forecasts (re-forecasts) of the past has been generated. This forms the basis of the following analysis. The System 4 re-forecasts were started every calendar month over the 30-year period 1981–2010 by emulating real forecast conditions when no observed information about the future is available at the beginning of the forecast. Here, we analyse (51 member) ensemble re-forecasts initialized on 1 May and 1 November 1981–2010. The forecast lead time is two to four months corresponding to the boreal summer (June, July and August, JJA) and winter (December, January and February, DJF) seasons.

In this study, we concentrate our analyses on 2 m temperature and precipitation over 21 standard land regions [17]. The verification data used are ECMWF re-analysis data (ERA-Interim) for 2 m temperature [18] and GPCP for precipitation [19]. As discussed in the Introduction, in seasonal forecasting, one is mostly interested in seasonal deviations from the long-term climatological mean. Observed anomalies for each year and season are defined as deviations from the mean over the 1981 to 2010 re-forecast period. In a similar way, model anomalies for each ensemble member were derived from the re-forecasts and the model mean over the re-forecast period. In order to emulate real-time forecast situations as closely as possible, both observed and model anomalies are computed in cross-validation mode by leaving out the actual forecast year in the estimate of the climatological mean values. Transforming absolute temperature and precipitation forecast values into anomalies implicitly also removes any systematic errors, or biases, which develop during the forecasts between the model and the verification.

In the following, we consider dichotomous, or binary, events *E* based on terciles of the climatological distribution of seasonal anomalies of temperature and precipitation. If *E* is defined as falling into the lower third of the long-term distribution, the event is called ‘cold’ for 2 m temperature or ‘dry’ for precipitation. Similarly, if *E* relates to the upper third of the distribution, the event is called ‘warm’ or ‘wet’. The seasonal forecasts from System 4 then specify the probability of event *E* that the seasonal-mean forecast anomalies lie below the lower tercile or above the upper tercile, respectively. Our aim here is to quantify the reliability of such probabilistic tercile events by comparing the forecast probability for *E* with the corresponding observed frequency of *E* of the verifying observations.

## 4. Reliability diagrams and categories of reliability

Reliability (or attributes) diagrams are tools to display and quantify the statistical reliability of a forecasting system, as defined in the Introduction. Such a diagram graphically summarizes for a given binary event *E* the correspondence of the *forecast* probabilities with the *observed* frequency of occurrence of the event *E* given the forecast. Reliability is high if this correspondence is very good. Reliability is poor if there is little, no or even negative correspondence between the forecast probabilities and the observed frequencies.

For example, suppose the seasonal forecast probability for event *E* is equal to 0.8. Then, in a reliable seasonal forecast system, *E* would actually occur, taking into account sampling uncertainty, on approximately 80% of the cases where *E* was predicted with a probability of 0.8. A reliability diagram displays a range of such forecast probabilities for *E* and their corresponding observed frequencies collected over the re-forecast period. If the correspondence between the forecast probabilities and the observational frequencies were perfect (and neglecting sampling uncertainty), then all data points would lie on a straight diagonal line in the reliability diagram. It is important to note that a forecasting system that always issues the underlying long-term climatological probability of the event has perfect reliability even though it might not provide any additional information to the climatological one. Such a forecast would result in just one point in the reliability diagram exactly at the climatological forecast probability and observed frequency of the event.

**Figure 1.**
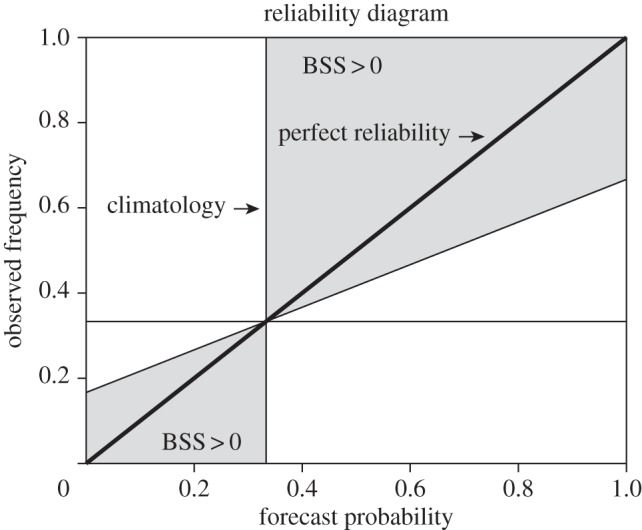
What is a reliability diagram? A reliability diagram shows the observed frequencies of an event as a function of its forecast probability. The thick diagonal line indicates perfect reliability. The thin horizontal and vertical lines show the climatological probabilities of the event in the forecasts and observations (here one-third for tercile events). The grey area defines a region in the diagram where data contribute positively to the Brier skill score.

Figure 1 shows a schematic of a reliability diagram for tercile events *E* but without any data points. Here, the climatological forecast probability and long-term frequency of *E* in the verification data (by definition one-third) are denoted by the vertical and horizontal lines. The grey areas in the diagram are linked to forecast situations where the Brier skill score^1^ (BSS), based on a no-skill climatological reference, is positive. What does this mean? Often, it is of interest to compare seasonal forecasts generated by physical models of the climate system with a reference forecast that serves as a benchmark for the climate model forecasts. Such a comparison allows the definition of skill scores of the forecasts: a skill score is positive (negative) if the forecast is better (worse) than the reference forecast. A widely used reference forecast is the simple climatological long-term mean forecast. For tercile events, the reference forecast would always be one-third. It can be shown [21] that forecast probabilities which fall into the grey indicated areas in figure 1 contribute positively to the BSS if climatology is used as a reference. The line that separates the skilful from the unskilful forecast probabilities is defined by BSS = 0 indicating that the forecasts below this line are not better than the reference forecast.

For any real forecasting system, the data points in a reliability diagram are not likely to lie on a straight line. In order to quantify the overall reliability of an event *E*, and to try to minimize the effects of relatively small statistical samples in estimating reliability, we apply a weighted linear regression as a best-fit estimate on all data points in the reliability diagram using the number of forecasts in each probability bin as weights. The slope of the so derived reliability line can be used as a quantitative measure of the reliability of the system: a slope of approximately 1 indicates very good reliability; a slope of approximately 0 a very poor or no reliability. A slope which is negative could be characterized as ‘worse than useless’ as it might encourage decision-makers to make decisions that could turn out to be exceptionally poor because of the inverse relationship between the forecast and observed probabilities. It is this slope of the reliability line on which our proposed categories of reliability will be based.

In this study, we use a definition of the binary events *E* that is based on thresholds of the lower and upper terciles of the climatological distribution. By construction, such a percentile-based event definition corrects biases, so that the climatological frequencies from both forecasts and observations are the same. An implication of this event definition is that the weighted linear regression reliability line always goes through the climatological intersection (one-third in our case). Different situations can arise for other event definitions. For example, fixed absolute thresholds of precipitation are often used for in the verification of weather forecasts. Here, it is, in principle, possible that the reliability line has a perfect slope of approximately 1 but is off-set from the diagonal. Such a situation reflects (unconditional) bias in the forecasts (see [22]).

In addition to the best-guess reliability slope, we estimate the uncertainty around that slope. Using a bootstrap algorithm with replacement, we draw randomly from the set of System 4 re-forecast data and compute the slope of the reliability regression line. By repeating this procedure 1000 times, we construct a re-sampled dataset of regression line slopes and use the 75% confidence interval of the resampling distribution to define an uncertainty range around our best-guess reliability slope.

**Figure 2.**
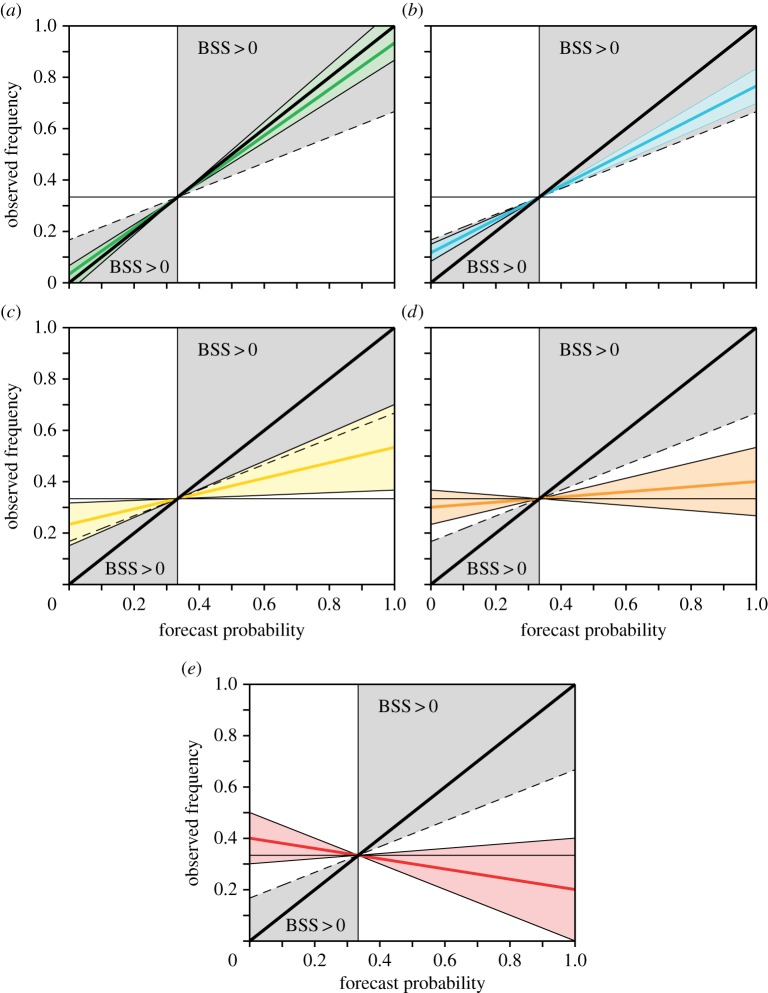
Five categories of reliability: (*a*) 5, perfect; (*b*) 4, still very useful for decision-making; (*c*) 3, marginally useful; (*d*) 2, not useful and (*e*) 1, dangerously useless.

**Figure 3.**
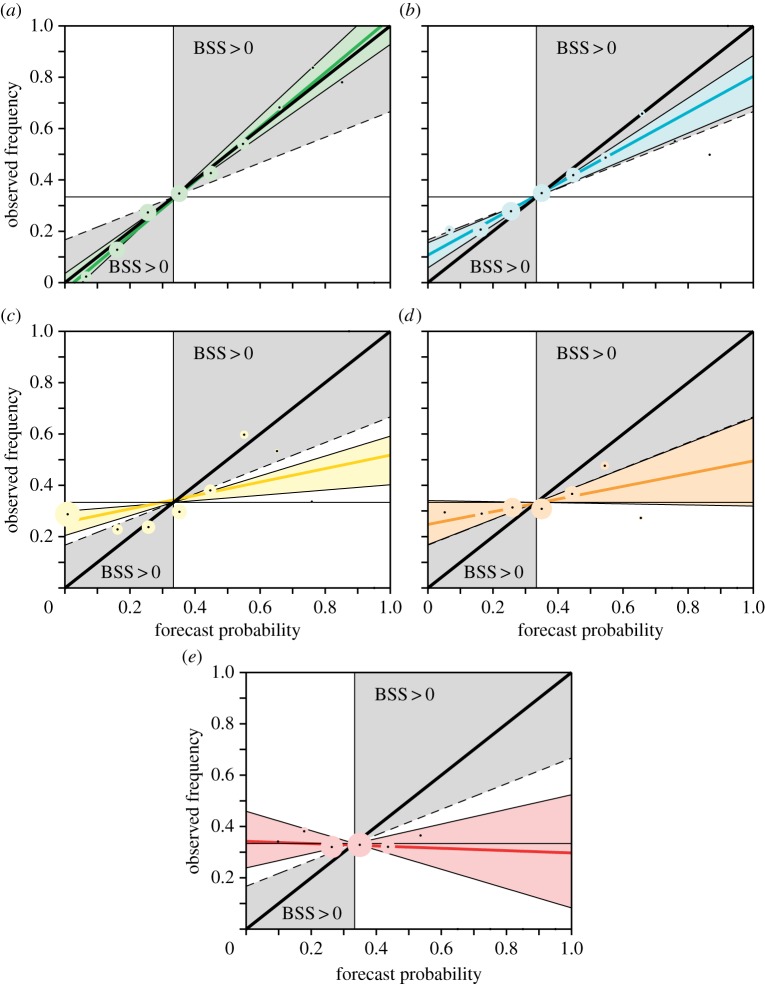
System 4 examples of the five categories of reliability: (*a*) 5, warm DJF over the Sahel; (*b*) 4, wet JJA over East Africa; (*c*) 3, dry DJF over West Africa; (*d*) 2, dry JJA over Southern Europe and (*e*) 1, dry JJA over Northern Europe.

In order to answer the question posed in the Introduction—how ‘good’ on a scale of 1–5 are our current seasonal forecasts—we propose a categorization of reliability based on the slope of the reliability line and the uncertainty associated with it. In figure 2, we show schematics for each of the five categories to demonstrate their definitions; figure 3 has examples for each category from the System 4 re-forecasts data. Here, the size of the data points is proportional to the number of forecasts in that forecast probability bin.

The highest *Category 5* classifies perfect reliability conditions (figure 2*a*). It is defined such that the uncertainty range of the reliability slope includes the perfect reliability slope of 1 and falls completely into the skilful BSS area. Thus, given the sampling uncertainty, such forecasts are perfectly reliable. Forecasts in category 5 can potentially be very useful for decision-making. In figure 3*a*, we show as an example for category 5 forecasts the reliability diagram for the tercile event of warm DJF over the Sahel region of System 4. Here, the best-guess reliability line is only slightly steeper than the diagonal. The uncertainty range clearly includes the perfect reliability slope of 1 (diagonal).

The second highest *Category 4* is characterized by reliability diagrams where the uncertainty range of the reliability line has at minimum a slope of 0.5 and does not include the perfect reliability line, see schematic in figure 2*b*. It describes forecast reliability that is still very useful for decision-making. An example from System 4 is given in figure 3*b* for wet conditions in JJA over East Africa.

If the slope of the reliability line is significantly positive but does not fall into either category 5 or 4, then the forecasts are classified as *Category 3* reliable (figure 2*c*). Such forecasts can be considered marginally useful for decision-making as they carry a partial positive relationship between the model forecast probability and the observed frequency of occurrence of that event. The signal of this relationship, or correlation, may be small but could still potentially be useful for some applications. As an example for System 4, dry DJF forecasts over West Africa as shown in figure 3*c* fall into Category 3 reliability.

If the slope of the reliability line cannot be distinguished, within its uncertainties, from zero, then the forecasts are defined as *Category 2* (figure 2*d*). Because of the approximately flat reliability line, there is no relationship between the forecast probabilities and the frequencies of the observed event; the forecast system is not useful for decision-making. An example from System 4 for Category 2 forecasts are the predictions of dry summers (JJA) over Southern Europe (figure 3*d*).

The poorest category of forecast reliability, *Category 1*, summarizes forecasts where the reliability line has a negative slope implying an inverse relation between the forecast probabilities and the frequencies of the observed event (figure 2*e*). These forecasts are dangerously useless for decision-making because they not only provide no useful information, but also can mislead the users of the forecasts with unreliable information. Dry summer (JJA) forecasts for Northern Europe from System 4 fall into this very unreliable category (figure 3*e*).

In principle, the raw probabilistic output from such seasonal forecast systems can be calibrated empirically using a training sample of data [22]. However, with limited training data (30 years is not a large sample), such empirical calibration cannot be assumed to produce reliable out-of-sample probability forecasts. As such, a key aspiration of any operational forecast centre must be to produce reliable forecasts without recourse to empirical calibration.

## 5. Reliability of System 4

This section gives a summary of how reliable the System 4 seasonal forecasts for near-surface temperature and precipitation in JJA and DJF are in terms of our five categories of reliability as defined in §4.

**Figure 4.**
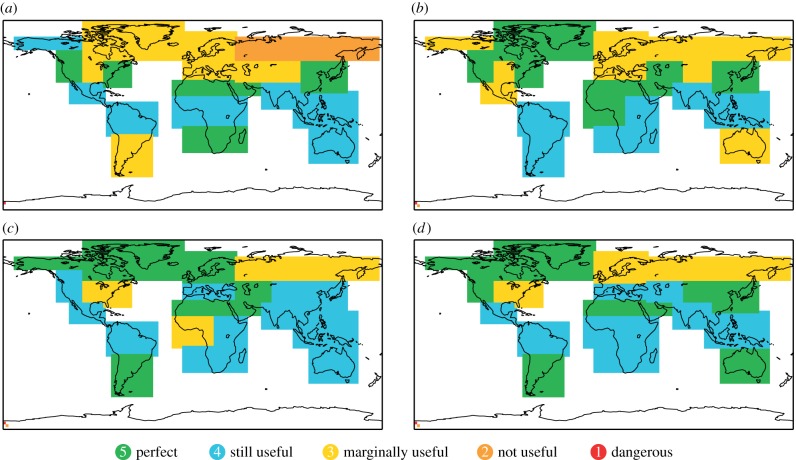
Reliability of System 4 seasonal forecasts for 2 m temperature. (*a*) Cold DJF, (*b*) warm DJF, (*c*) cold JJA and (*d*) warm JJA.

In figure 4, we show the reliability categories for 2 m temperature in DJF and JJA over 21 land regions around the world. Almost all of the areas fall into the first three categories from perfect to marginally useful reliability. Only the Northern Asia region for cold DJF events has been classified as not useful (Category 2).

Remarkably, there are a number of extra-tropical regions where reliability is found to be perfect (Category 5): the east and west coasts of North America and parts of China and East Asia in DJF, and South America, Southern Africa and Australia in austral winter in JJA.

For Europe, all winter predictions of temperature fall into the marginally useful Category 3, whereas in summer the temperature forecasts over Europe is improved. Cold anomalies over Northern Europe are classified to have perfect reliability. Category 4 reliability of still being potentially very useful after calibration has been found for Southern Europe and Category 3 performance is shown for warm summers over Northern Europe.

Over the extended tropical areas, temperature forecasts in both seasons are classified as either Category 5 or Category 4, except for cold anomalies over western tropical Africa in JJA which have Category 3 reliability. The Sahara region is an area that consistently falls into Category 5 for cold and warm temperature events in JJA and DJF.

**Figure 5.**
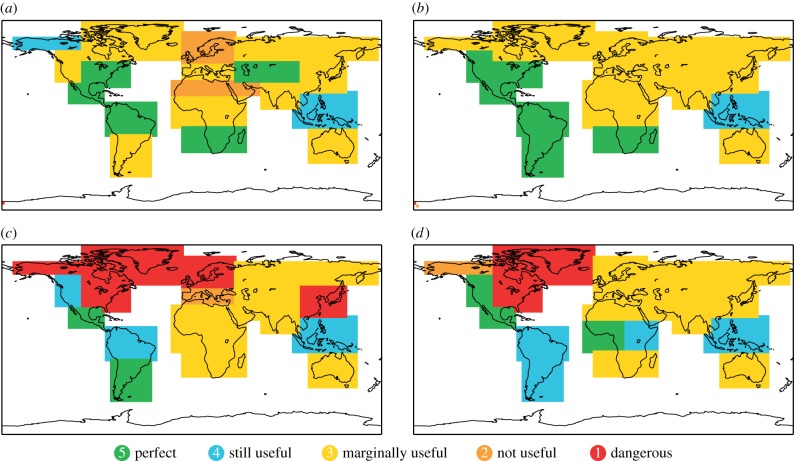
Reliability of System 4 seasonal forecasts for precipitation. (*a*) Dry DJF, (*b*) wet DJF, (*c*) dry JJA and (*d*) wet JJA.

Results for the reliability categorization of precipitation forecasts are shown in figure 5 for wet and dry DJF and JJA seasons. Overall, the reliability performance for precipitation is poorer than for temperature with more regions being classified with lower categories. The marginally useful Category 3 is the most frequent category for precipitation forecasts in both seasons and for both wet and dry events.

Even though the overall performance is not very reliable, there are areas and events that are classified as perfectly or usefully reliable. Consistent regions among the events and seasons are Central America, Northern parts of South America and southeast Asia.

Over Northern Europe, the reliability of precipitation forecasts for winters (DJF) is not useful (Category 2) for dry events and marginally useful for wet events. Southern Europe falls into the middle Category 3 in winter and for the wet summer events. The reliability for dry summers over Europe is notably poor with Southern Europe classified as not useful (Category 2) and Northern Europe as dangerously useless (Category 1). Parts of Northern America also fall into that lowest category.

**Figure 6.**
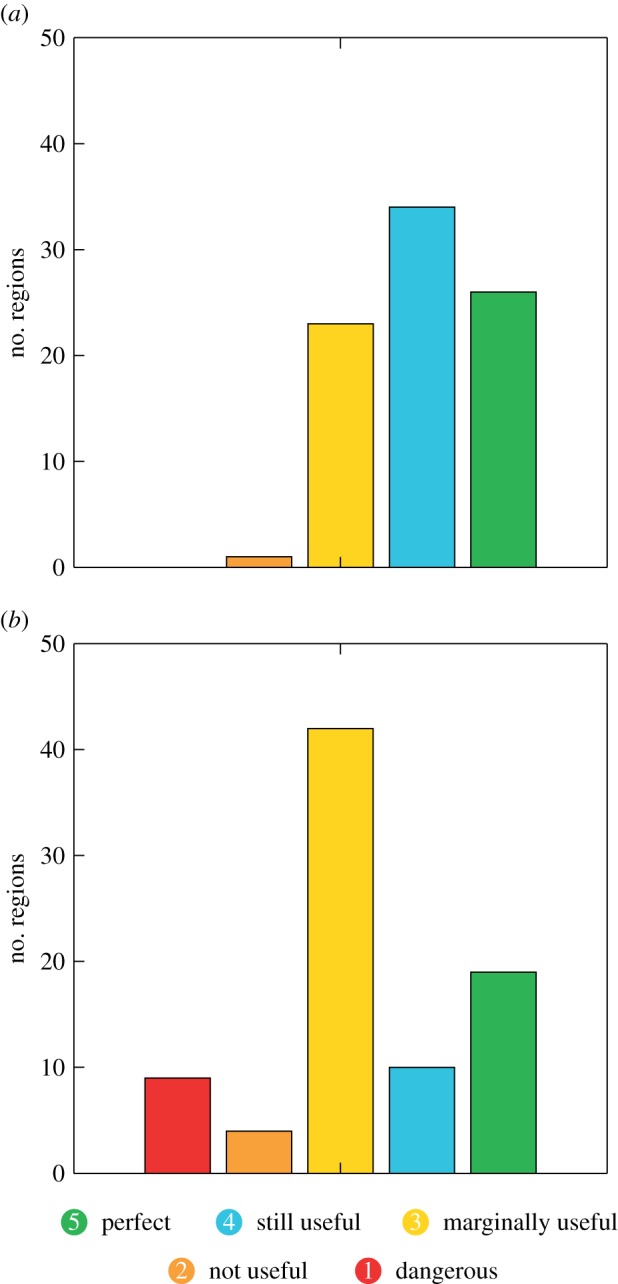
Number of regions that fall into each reliability category summed over all four events for (*a*) temperature and (*b*) precipitation.

To summarize these findings, figure 6 shows how many regions there are in each of the five reliability categories when accumulated over all seasons and tercile events. The most frequent category for the temperature forecasts is Category 4, describing forecasts with good reliability that can still be useful for decision-making. The perfect reliability Category 5 has also been found for a lot of regions while only one of the regions fell into the not useful Category 2. None of the temperature forecasts was classified as dangerously useless in terms of its reliability.

As mentioned above, the most frequent reliability category for precipitation is the marginally useful Category 3. Such forecasts are not very reliable, but might potentially be marginally useful for some applications. The category with the second highest number of regions is the one of perfect reliability, which is an optimistic result for the usefulness of seasonal forecasts of precipitation. However, there are substantially more cases of areas that have poorer reliability than there are for temperature (Categories 1 and 2); users should not use these forecasts for decision-making in these regions as they can be dangerously misleading.

## 6. How can seasonal forecast reliability be improved?

The above-mentioned results suggest that we still have some way to go before it can be said that the goal of providing users with reliable forecasts has been achieved, particularly for precipitation and away from the El Niño region. There can be little doubt that the ability to understand and represent physical processes accurately is key to improved reliability.

In a recent study based on integrations within the project Athena [23], Dawson *et al.* [24] were able to show in AMIP (Atmospheric Model Intercomparison Project) integrations that the ECMWF model could simulate the non-Gaussian structure of observed Euro-Atlantic weather regimes more accurately in a model with spectral resolution T1279 (approx. 15 km) than with resolution T159 (approx. 125 km). It is plausible that the improved simulation of such weather regimes in a T1279 model is associated with better representation of topography on the one hand, and with a more realistic representation of Rossby wave breaking on the other hand.

Improved simulation of stratospheric processes through finer vertical resolution is also expected to impact seasonal forecasts [25,26]. Other potential processes to improve the seasonal predictability include sea-ice or snow cover over the Eurasian land areas.

A fundamental question, but one that is probably unanswerable until the tools are available to answer it, is whether perfect forecast reliability can only be achieved when convective cloud systems (with scales of just a few kilometres) are resolved explicitly. Much of the skill of seasonal forecasts originates in the tropics, and moist convection is a dominant form of instability in the tropics. Seasonal forecasting with such cloud-resolved models will require exascale computing capability.

A better representation of other Earth system components is also likely to improve reliability. For example, Weisheimer *et al.* [27] showed that a better representation of land surface processes led to remarkably good probabilistic forecast of the summer 2003 heatwave.

On the other hand, because the climate system is chaotic, it is necessary to represent inevitable uncertainties in the representation of processes which have to be parametrized. A programme to represent parametrization uncertainty has been ongoing for some time at ECMWF [28–30] and was shown to reduce some of the biases related to tropical convection in System 4 [31]. On the monthly and seasonal timescales, there is evidence that it is competitive with, and for temperature predictions can outperform, the more standard multi-model ensemble approaches to the representation of model uncertainty [32]. Furthermore, improved initial conditions based on higher quality and quantities of observations are also vital for the reduction of model error.

There can be little doubt that the value to society of reliable non-climatological predictions of seasonal climate. However, to develop a high-resolution system with accurate stochastic representations of model uncertainty in all relevant components of the Earth system, is not only a formidable technical challenge, it may be one that will require computing resources that are unavailable to individual institutes in the foreseeable future. A possible route to achieve the goal of a reliable seasonal climate prediction system, based on much stronger international collaboration, has been presented elsewhere [30,33–35].

## 7. Conclusion

Let us return to the question posed in the Introduction. What constitutes a ‘5’, to which a seasonal forecast system should aspire? Here, we propose the following broad criterion for rating a seasonal forecast system a ‘5’: when the system predicts probabilities *ρ*(*X*) that are substantially different from the climatological distribution *ρ_C_*(*X*), then these probabilities can be relied on, and acted on by decision-makers. Note that we make no firm statement about how often such situations arise. It may be that in the majority of cases the forecast system does not predict probabilities that differ substantially from *ρ_C_*(*X*). If this is the case, then the probabilistic skill score may not differ substantially from zero. However, for such a forecast system, a user does not make a bad decision based on unreliable forecast information.

At this stage, it remains to be demonstrated how our proposed categories of reliability will be used in real life decision-making. In the light of the increasing need to develop a dedicated forecast–user interface, we refer the reader to a new interdisciplinary project in which the authors are involved (funded under the Oxford Martin Programme On Resource Stewardship) explicitly addressing the utility of probabilistic seasonal forecasts (http://www.oxfordmartin.ox.ac.uk/institutes/resource_stewardship/).

Reliability of seasonal forecasts can also be considered relevant in the context of seamless prediction of weather and climate: the reliability of climate predictions on the seasonal timescale can provide constraints for the trustworthiness of climate change projections. Reliability diagrams provide a means to calibrate climate change probabilities and discount these climate change probabilities if the seasonal forecasts can be shown to not be reliable [36].

The ECMWF seasonal forecast System 4 cannot be rated a ‘5’ for all regions of the world, and for all variables. We have shown that for surface temperature, and even more for precipitation, forecast probabilities are not reliable when different from climatology and away from the El Niño region. Based on current performance our current capability to forecast seasonal climate was rated between 1 and 5 depending on variable and scale. However, given expected increases in resolution, and better stochastic representations of model uncertainty, we see no reason why this should not rise to 5 overall in the coming 30 years.

## Endnote

1 The BSS is based on the Brier score [20] which can be considered the probabilistic generalization of the mean-squared error for dichotomous events.
